# Assessment of Cognition and Language Using Alternative Response Modalities

**DOI:** 10.1177/10731911251315012

**Published:** 2025-02-12

**Authors:** Kristine Stadskleiv, Katy Latham, Kristina Tufteskog Spanne, Karen Sætre, Anna Fraas, Ilaria Ruscito, Yasmine Taha, Janice Murray

**Affiliations:** 1 University of Oslo, Norway; 2 Oslo University Hospital, Norway; 3 Manchester Metropolitan University, UK

**Keywords:** adaptation, assessment, cerebral palsy, cognition, intelligence, motor impairment, test

## Abstract

Assessing cognition and language using standardized tests is challenging when the individual has severe speech and motor impairments. Tests with a multiple-choice format may be adapted without compromising standardization using alternative response modes like partner assisted scanning (PAS) and eye-pointing (EP). Standardization of such assessment is little researched. The study investigates the (a) reliability of, (b) transparency of, and (c) adherence to assessment protocols using PAS and EP. The participants were students from special needs education and speech and language therapy, who worked in dyads (*n* = 39). Two observers recorded a number of errors made in protocol delivery, independently of each other. The dyads made between 0 and 81.5 errors. Number of errors was not related to response mode, *t*(38) = –0.21, *p* = .839. The observers were in high agreement, with an intraclass correlation coefficient of .97, *p* < .001. The study suggests that assessing language involving alternative modes of responding can be successfully taught to novice practitioners.

Assessments of cognition and language rely heavily on the use of normed tests (Strauss et al., 2006). These tests should be administered in the specified manner for the norms to be applicable. Although the assessments are assessments of the mind, a person’s cognitive and linguistic capabilities are inferred from some form of action or performance executed by the person assessed. This might involve drawing, writing, speaking, building, and often in as rapid a manner as possible (Anderson et al., 2008). Even tests placing minimal demands upon fine motor abilities, such as tests with a multiple-choice format, require a method of indicating the chosen answer option. Typically, this is done by finger-pointing to the chosen answer option.

For some persons, even this minimal motor involvement is not possible. Persons with very severe motor impairments have limited ability to control their body movements and to use motor speech movements to communicate effectively in a way that demonstrates their cognitive-linguistic abilities, and even multiple-choice tests require fine motor skills they do not have. Such severe motor impairments may be due to medical conditions like cerebral palsy (CP), acquired brain injuries, motor neuron diseases, locked-in syndrome, and developmental progressive neuromuscular diseases, among many others. In CP, which has a prevalence of 2 in 1,000 live births (Hollung et al., 2018), approximately one-fifth of this population (Andersen et al., 2022) has such severe fine motor impairments that they will struggle with finger-pointing, that is, they are classified at levels IV and V on the Manual Ability Classification System (MACS; Eliasson et al., 2006). The prevalence of other conditions, such as motor neuron disease (8 per 100,000) and head/brain injury (300 per 100,000, where approximately 20% have so severe injuries that they require alternative forms of communication to speech), and locked-in syndrome (2 per 100,000), indicate that around 1 per 1,000 have so severe motor impairments that they will struggle using finger-pointing as response modality on psychological tests (Creer et al., 2016). Although these might seem like marginal populations, the importance of accurate assessments for the individuals involved cannot be overstated.

First, to achieve their potential, children and adults with severe speech and motor impairments are in need of interventions. These might include augmentative and alternative communication (AAC), general assistive technology, and educational or employment support. AAC comprises of linguistic expressive modes such as manual signs, graphic symbols, and text and is used by people unable to use speech as the main mode of communication (Beukelman & Light, 2020). They may also have cognitive impairments, for which they require interventions. For these interventions to be helpful, knowledge about the characteristics of the individual’s cognitive and language functioning is needed.

Second, the cognitive functioning in this group varies from profound intellectual disability to age appropriate or better (Geytenbeek et al., 2015; Stadskleiv et al., 2018). It is not possible to reliably estimate cognitive functioning based on the degree of motor impairment, as there is no one-to-one correspondence between the two (Blair, 2010; Sigurdardottir et al., 2008). Individual assessments of cognition and language are therefore necessary.

Finally, many children and adults with severe speech and motor disorders benefit from using communication aids to support their participation, improve their quality of life, and have better mental health outcomes (Romski et al., 2015). However, communication aids are sometimes recommended without full language assessments, contributing to the person not using the aids they have been given (Lynch et al., 2019; Moorcroft et al., 2019; Murray et al., 2021). A full assessment of language and cognition reveals persons’ particular strengths and challenges (Strauss et al., 2006). Without this, their abilities may be under- or overestimated and the supports provided may not be the best fit (Stadskleiv, 2015). Unused communication aids mean that children and adults cannot achieve their potential. The gravity of inappropriate or insufficient assessment might have on an individual’s life is illustrated by a documented case where an individual, before assessment, was considered to have a profound intellectual disability and not offered AAC. The woman participated in a research project on communication with adults whom her next-of-kin struggled to interpret. As part of the project, cognition was assessed. After this assessment, it was discovered that the woman, who was in her mid-50s, only had a moderate intellectual impairment and was in urgent need of a complex AAC system (Kildal et al., 2021).

Despite the need for assessment of children and adults with severe motor impairment, they are often not assessed appropriately. Studies from Norway and Sweden, where all children with CP should be offered cognitive assessments, have found that the part of the population with the most severe motor impairments is often not assessed (Knudsen et al., 2022; Påhlman et al., 2019; Stadskleiv et al., under review). One reason why assessment is not done may be the perceived lack of appropriate tests (Geytenbeek et al., 2010; Smith, 2015). Tests that require the person to name or point to one or more items from an array of possibilities in response to adult prompts, like “show me the blue car,” is a method of response not possible for children or adults with complex motor disorders (Geytenbeek et al., 2010). As a result, available tests are rarely used with these children, with many receiving support based only on estimated abilities (Stadskleiv, 2020), that is, cognitive or language functioning is assumed, but not assessed.

Positively, it is not necessary to assume functioning as it is possible to use standardized tests with a multiple-choice format applying other means of indicating the answer than finger-pointing (Fiske et al., 2020; Kurmanaviciute & Stadskleiv, 2017). This involves using alternative response modalities such as partner assissted scanning (PAS) and eye-pointing (EP). In PAS, the assessor points out the alternatives systematically and the person tested responds by confirming or rejecting each alternative in turn (Kurmanaviciute & Stadskleiv, 2017). EP refers to the controlled and intentional use of eye-gaze, that is, the child or adult’s use of purposive gaze fixations and shifts between a response item and the assessor, so they can draw the assessor’s attention to their choice (Clarke et al., 2020; Sargent et al., 2013). It is possible to assess both language comprehension and cognition using such alternative response modes (Kurmanaviciute & Stadskleiv, 2017).

A small body of research has compared the effect of alternative response modes to standardized responses. Eye-tracking technologies were used in studies examining EP as a response modality in assessment with older preschool children and school-aged children (Fiske et al., 2020; Kurmanaviciute & Stadskleiv, 2017) and adults (Thurén, 2010). Paper-based tests have been used to investigate to use of alternative response modes in school-aged children, comparing finger-pointing to EP (Spillane et al., 1996) and to PAS (Arvidson, 2000). This research suggests that assessment outcomes are not significantly affected by response modality in typically developing populations. However, these studies are small in scale (*n* = 27–48). Furthermore, they do not include all age groups.

## Research Questions

Although there is support for alternative response modes, there is currently no clear protocol available to ensure that assessors use a consistent approach during assessments. Therefore, the aim of this study was to investigate the (a) reliability of, (b) transparency of, and (c) adherence to protocols for assessments using alternative response modes, specifically evaluating the response modalities PAS and EP. The assessment protocols were presented in two formats, one with written instructions and one with video instructions. To evaluate if the protocols were transparent and easily understood by novice practitioners, they were piloted with students who were being trained in the assessment of cognition and language. By trying out the protocols on students and not on trained professionals, we ensure that the protocols are transparent enough to be understood by professionals with limited experience in assessing clients with severe speech and motor impairments.

## Method

This study has a cross-sectional design, with two cities of data collection (Oslo, Norway and Manchester, UK).

### Participants

Participants comprised three groups: (a) observers, (b) assessors, and (c) responders. The observers partook in the design of the study and evaluated the performance of the assessors. The assessors were the students administering the tests, while the responders were students or professionals familiar with EP/PAS techniques who responded to the test items using the response modalities PAS and EP.

There was a total of 10 observers, working in pairs. Six of the observers were students (four master students from the Department of Special Needs Education at the University of Oslo (UiO) and one undergraduate student and one master student in Speech and Language Therapy from the Department of Health Professions at the Manchester Metropolitan University (MMU)), and four observers were SLP researchers from MMU, of which three were Senior lecturers and one Professor. The students who acted as observers worked as research assistants on the project.

Assessors were students, that is, novice practitioners. Power calculations prior to enrollment indicated that to detect performance above chance level (defined as over 50% correct), when alpha is set at 0.05 and power at 95%, each group should comprise at least 16 participants. As we wanted to compare two modalities (EP and PAS) and two formats (written and video instructions), we aimed to recruit at least 32 dyads. From UiO, we recruited 40 students who worked in pairs, thereby comprising 20 dyads where one student was the assessor and the other the responder. At MMU, 19 students worked together with a professional who role-played (simulated) being a child assessed, thus rendering 19 dyads. The total sample of assessors and responders therefore comprised 39 dyads.

Inclusion criteria for the assessors were (a) being an undergraduate or master student of special needs education or speech-language therapy at either UiO or MMU and (b) having received some basic introduction to assessment, that is, being novice practitioners. At UiO, this made all students of special needs education at the master level eligible, and assessors and responders were recruited via digital sources and opportunity recruitment during university-based teaching. At MMU, undergraduate and graduate students with at least one clinical placement and thus some experience of typical assessment were eligible. Students with no experience of language assessment and class-based training were not invited to participate as assessors.

### Instruments

**Figure 1.**
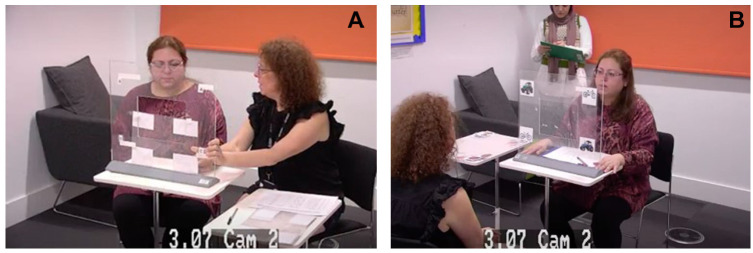
Set up Using (A) Partner Assisted Scanning (PAS) and (B) Eye Pointing (EP) (Examples With Research Staff and Research Students Included in the Images, With Consent).

Investigation of the response modalities was done using paper-based test materials, meaning that EP was in the form of observation of looking behaviors, not using powered-technological solutions (Figure 1B). Specifically, the assessor held up the assessment material at eye height, with stimulus items placed in each corner of a laminated see-through material. They then observed which of the four answer options the responder was looking at (see Supplemental Material for an illustration). Although electronic eye-gaze technology offers many possibilities, there are at least three reasons from a clinical point of view for why it is important to also have a test in paper-based formats. First, research into typically developing children younger than four years of age suggests that they often struggle with the attentional and physical demands of eye-tracking technology calibration, which is required for accurate measurement (Clarke et al., 2020; Griffiths, 2020). Second, it can be challenging to use technological solutions for those with eye-motor impairments (Stadskleiv, 2015). Third, technological tools such as eye-tracking technology are not available for all clinicians, as they are expensive.

The material used in the study was developed specifically for the study, as there is no commercially available paper-based test suitable for both EP and PAS. The material consists of five components: (a) written information about alternative response modes, (b) instructional protocols, (c) language task sets, (d) assessment scoring forms, and (e) observation forms.

#### Written Information About Response Modes

To offer orientation to the aim of the study, the assessors were provided with a page of written information about the alternative response modes EP and PAS.

#### Instructional Protocols

The assessors were provided with detailed protocols for administration with an alternative response mode, that is, one instructional protocol describing EP and one instructional protocol describing PAS. Both instructional protocols had two formats, that is, a written protocol and a video protocol. The written protocols gave a detailed explanation of how to administer the test. That included a visual example on how to execute a test item and the wording for what to say. In the video protocol, it was explained how to administer the test simultaneously with a visual demonstration of either PAS or EP delivery.

#### Task Set

A set of language test items were created specifically for this project. The task set included a set of 40 items, assessing comprehension of spoken words and simple sentence structures, using a multiple-choice format where four answer options are presented per item. The words and sentences were chosen from the Norwegian version of the McArthur-Bates Communicative Development Inventory (Kristoffersen & Simonsen, 2012). This comprised the first vocabulary typically acquired by children under 4 years of age. This was done so we could evaluate response modality, not language comprehension. Furthermore, weight was placed upon choosing graphic illustrations that were clear and visually accessible.

**Figure 2.**
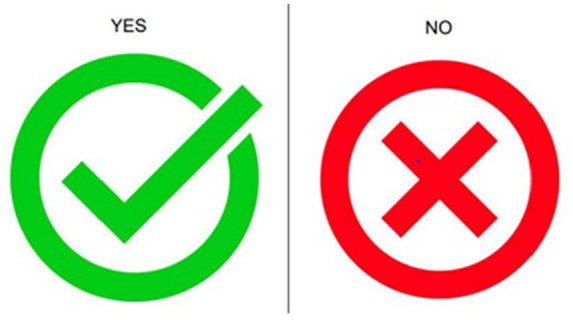
Graphic Symbols for YES and NO for PAS as Mode of Administration.

**Figure 3.**
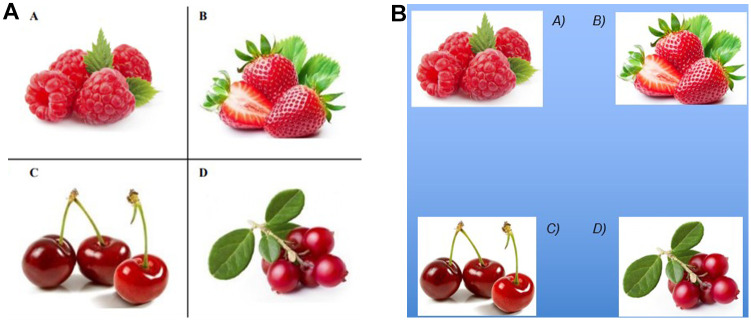
Illustrations of Items Used to Assess Vocabulary Comprehension, With PAS and EP. *Note.* (A) Example of item used to assess comprehension of the word strawberry (answer Option B) with PAS as response mode. Each answer option (A to D) is pointed out by the assessor, simultaneously asking “is it this one?” to which the responder could reply YES or NO. (B) Illustration of item used to assess comprehension of the word strawberry (answer Option B) with EP as response mode. The answer options are presented on a laminated transparent sheet, which makes it possible to see through. The respondent points out an answer option by looking at it.

The person being tested (the responder) should show their understanding of the spoken word or sentence by indicating one of four pictures. When using PAS, the assessor at UiO provided graphic symbols for *YES* and *NO* (see Figure 2) in addition to the item (see Figure 3A), so that participants could accept or reject the answer options as they were pointed out to them. At MMU, the responders indicated “yes” and “no” nonverbally, for example, by nodding and shaking their heads or looking up or down, respectively. When using EP, an answer was indicated by looking directly at the answer option (see Figure 3B).

#### Assessment Scoring Form

Both for EP and PAS, the respondents’ answers were noted down in an assessment scoring form by the assessors.

#### Observation Form

The observers rated the assessors and responders as they completed the task sets, using an observation form. The observations noted included the time the assessments took and the type and frequency of any errors in administration.

### Procedure

In Norway, the students worked together in pairs, and it took around 30 minutes per dyad. Beforehand, the observers randomly chose which of the participants would try out the protocol on the other student (the assessor) and who should play the role of a child being assessed (the responder). In the UK, all recruited students were assessors, and they assessed a professional, who role-played being a child assessed. In both countries, the “child” did not always respond correctly, simulating real life and allowing an appraisal of how the novice assessor responded.

The assessor and the responder worked in dyads. The assessor in the dyad was not given a time limit to familiarize themselves with the protocols. The assessor received or viewed the protocols in the absence of the responder (“child”). The observers made sure that there was a systematic variation between giving EP or PAS first, and whether the protocol was presented in video or written format. For a subset of MMU dyads, it was decided to investigate the effect of providing the video and written protocols at the same time. Before proceeding with EP, the dyads practiced with a training item to check that the assessor interpreted the responder’s gaze direction correctly.

During the live test situation, there were two observers who scored the execution of the assessments, separately from each other. All sessions were filmed as a way of verifying observation accuracy.

Data collection first took place at UiO, during which a decision was made to make a few adjustments. After 12 dyads had been completed, two minor adjustments were made to the written protocol to help the participants execute the testing in a more precise way. The first adjustment was to add the sentence “complete the sequence regardless of which answer is correct,” to make it more clear that they should point out all four answer options for all items (in the first version it only said to “complete the sequence”). The second adjustment was a change in the instruction for PAS on how the person being assessed should confirm or reject an answer option. In the first version, it said, “Use the accompanying graphic symbols,” which was changed to “The person being assessed should point to the accompanying graphic symbols as answer options.” There were no changes made to the information in the video protocols, however, it was decided to show the videos two times to make sure the information was understood. Data collection at MMU commenced after data collection at UiO was completed, using the adjusted UiO version of the protocol.

### Analyses

Analyses involved a quantitative analysis of type and number of errors observed and the statistical analyses performed.

#### Error Analyses

The performance was analyzed in relation to what types of errors were made in the dyads during the assessment process. A total of 12 different types of errors were identified (see Table 1), and it was noted what types of errors were made and how many. Errors were noted for the dyads performing the tasks, as observed by the two observers independently of each other. The instructional protocols for EP and PAS informed the observers while noting down errors. The error typology was created using an inductive approach where the qualitative observations of errors made in the dyads, as noted by the observers, were categorized. For example, it says in the EP instructional protocol “Look directly at the person being assessed, not at the picture showing the correct answer option.” The observer noted down if the assessor looked at the correct answer option, and this was then coded as the assessor having a “leading gaze during assessment.”

**Table 1:** The 12 Types of Errors That Were Made During Assessment, With Specifications Pertaining to PAS and EP Modalities, as Relevant.

Type of error	Examples of errors	
Addressing the observer	Addressing the observer with questions or requests for clarifications during test administration	
Disruptions	Any disruption during the assessment, such as a mobile phone ringing	
Assessment scoring form not used correctly	Assessment scoring form either not used or not correctly filled out, thereby making scoring of task not possible	
Task not properly introduced	Task not introduced as described in written or video instruction	
Instructions incomplete	The responder asks what he/she is expected to do after the assessor has finished the task introduction	
Wrong phrasing of test question	Question for the item not properly asked or omitted. Should be completely asked, as per instruction, on at least the first three tasks	
	PAS	EP
Wrong placement of the assessor	Seated opposite respondent, making it difficult to point out answer options sequentially	Seated next to respondent, making it difficult to see gaze direction of respondent
Leading tone of voice during the assessment	Assessor puts emphasis when asking *is it this one?* while pointing out the correct answer option	Assessor puts emphasis on the word *this* when pointing to the correct answer options during the introduction of the four answer options
No use of yes/no symbols (used at UiO, not MMU)	Yes/no symbols for responding to answer options either not used or placed out of reach of the responder	(not applicable)
Leading gaze during assessment	(not applicable)	Assessor looking at correct answer option
Wrong placement of test material	(not registered)	Test material not placed at eye height and between assessor and responder
Incomplete procedure/sequence	Not pointing out all four answer options (must do this on all items)	Not pointing out all four answer options (should at a minimum be done on the first item)

#### Statistical Analyses

Descriptive statistics were used to provide an overview of the types of errors made during administration. Intraclass correlation coefficients (ICCs), that is, two-way mixed effects model where people effects are random and measures effects are fixed, were used to investigate the degree of agreement between observers, reporting also the 95% confidence intervals (CI). To compare the mode of instruction (i.e., written versus video instruction first), order of access (i.e., EP or PAS first and second), and effect of adjusting the instruction (i.e., pre and post text adjustment), independent samples t-tests were used. Paired-samples t-tests were used to investigate the number and types of errors made using EP and PAS. Cohen’s d effect sizes are reported, where 0.2 represents a small effect size, 0.5 a medium effect size, and 0.8 a large effect size (Cohen, 1988). Effect sizes for one-way ANOVA are reported as η^2^, where 0.01 indicates a small effect, 0.06 indicates a medium effect, and 0.14 indicates a large effect (Pallant, 2010). For the two dependent variables, “categories of errors” and “number of errors,” Levene’s test of homogeneity of variances was nonsignificant (*p* > .05) for the three groups (UiO dyads pre-adjustment of protocol, UiO dyads post-adjustment of protocols, and MMU dyads). The variable “categories of errors” was normally distributed, with *p* > .05 on both the Kolmogorov–Smirnov and Shapiro–Wilks tests. The variable “number of errors” was not, but with a sample size exceeding 30 parametric statistics may still be used (Pallant, 2010).

### Ethics

All participating students gave a written consent. The participants were informed that the purpose of the study was not to assess their language skills, but to evaluate the effectiveness of the training protocols and if a written or filmed protocol is preferable when introducing adapted response modalities in cognitive and language assessment. As the language test items chosen were uncomplicated and the pictures used in the task were clear, we did not anticipate that participation in the study should constitute a stressful situation for the participating students being “assessed.”

Ethical approval for the Norwegian part of the study was obtained from the Norwegian Center for Research Data (application #597598/2021). Videos of the assessment situation were stored at the UiO’s secure server for sensitive data (see TSD; tsd-drift@usit.uio.no). Ethical approval for the UK part of the study was obtained from Manchester Metropolitan University Ethics Review Committee (approval reference number 43270).

## Results

### Agreement Between Observers

**Figure 4.**
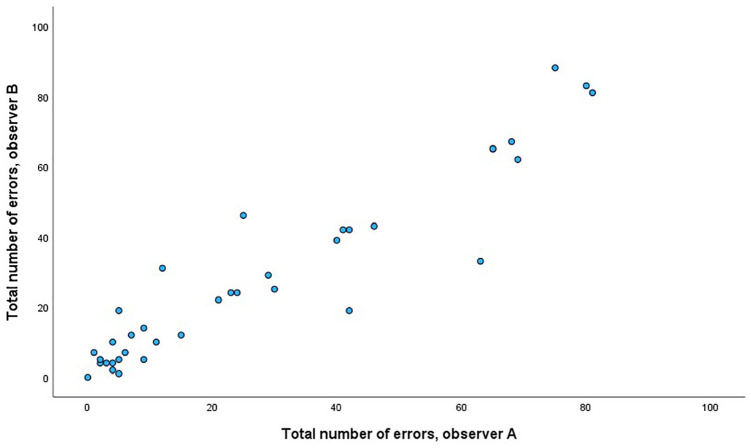
Number of Errors Recorded by Two Observers (A and B).

Intraclass correlation coefficients for the two observers (*n* = 37 dyads) were .97, 95% CI [.94, .98], *p* < .001, for total number of errors recorded; .97, 95% CI [.94, .98], *p* < .001, for errors recorded during PAS administration mode; and .95, 95% CI [.91, .98], *p* < .001, for errors recorded during EP administration mode (Figure 4).

### Descriptive Overview

In the 39 dyads, the number of errors recorded ranged from 0 to 81.5 (range 0–51 for EP and 0–62 for PAS), with a combined mean of 27.9 (25.5) for EP and PAS errors. The dyads made errors in on average 2.3 (ranging from 0–6) and 2.6 (ranging from 0–5) categories, a nonsignificant difference with a small effect size. Table 2 provides an overview over types and number of errors made in the 39 dyads during the assessment process and shows that there were no significant differences in the types of errors made using EP and PAS. The dyads spent on average 144.0 seconds (*SD* 48.5) to familiarize themselves with the instructions for using PAS and 137.4 seconds (49.1) for using EP, a nonsignificant difference, *t*(19) = –0.59, *p* = .564, *d* = 0.13 (this measure is only available for *n* = 20 dyads from UiO). There was no significant difference in time used to complete the assessment using EP (*M* = 502.6, *SD* = 33.6) or PAS (*M* = 538.3, *SD* = 27.7), *t*(29) = –1.2, *p* = .239, *d* = 0.22.

**Table 2:** Errors Made During Assessment Using PAS and EP Modalities in 39 Dyads, Mean (SD) for All Scores and Range for Overall Scores (Scores From Observers A and B Combined)

Type of error	PAS	EP	Sign^a^	Cohen’s d
Addressing the observer	0.5 (0.5)	0.5 (0.5)	n.s.	0.03
Disruptions during assessment	0.6 (1.2)	0.5 (0.7)	n.s.	0.14
Assessment scoring form not used correctly	0.2 (0.4)	0.2 (0.4)	n.s.	0.00
Task not properly introduced	0.3 (0.4)	0.2 (0.4)	n.s.	0.16
Instructions incomplete	3.1 (7.2)	2.3 (5.6)	n.s.	0.12
Wrong phrasing of test question	2.2 (5.1)	4.3 (7.5)	n.s	0.25
Wrong placement of the assessor	0.1 (0.3)	0.1 (0.2)	n.s	0.24
Leading tone of voice during assessment	1.5 (2.7)	1.4 (3.0)	n.s.	0.06
Wrong use of yes/no symbols	5.5 (8.9)		–	–
Leading gaze during assessment		0.6 (1.0)	–	–
Wrong placement of test material		0.1 (0.3)	–	–
Incomplete procedure/sequence	4.4 (8.0)	2.5 (5.0)	n.s.	0.23
**Overall**				
Categories with errors	2.3 (1.5) (0–6)	2.6 (1.3) (0–5)	n.s.	0.16
Total number of errors	13.7 (16.8) (0–62)	14.3 (15.0) (0–51)	n.s	0.03

a Paired-samples *t*-tests comparing mean number of errors using PAS and EP.

**Figure 5.**
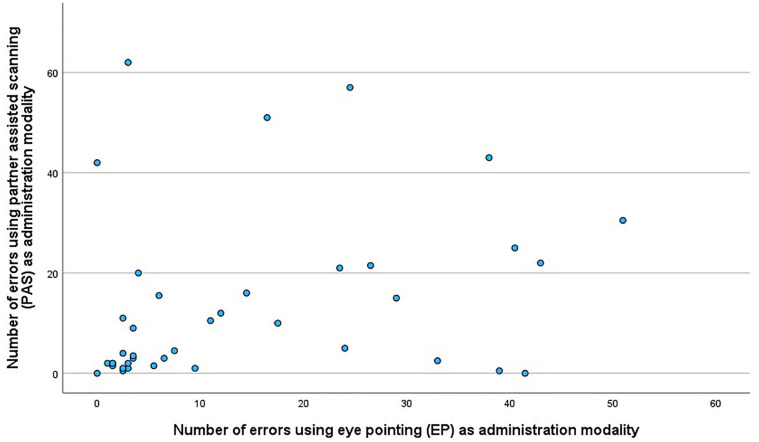
Relationship Between Number of Errors Made on PAS and EP in the 39 Dyads.

The error categories where the highest number of errors were made (up to 20 recorded for one dyad), were “incomplete instructions,” “wrong phrasing of test questions,” “wrong use of yes/no symbols,” and “incomplete procedure/sequence.” Only seven dyads made a significant number of errors (>20) at both PAS and EP (Figure 5). The relationship between the number of errors made using PAS and EP did not reach significance, *r* = .28, *p* = .087.

**Figure 6.**
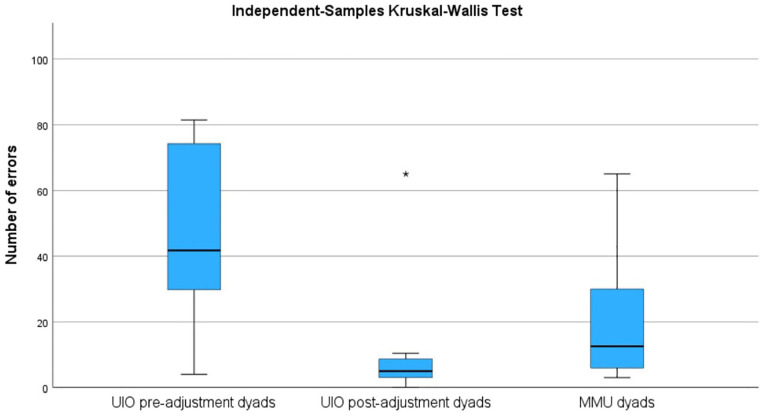
Number of Errors (Average of Two Observers) Made in Dyads From UiO Prior to Adjustment of Instructional Protocol (Pre-Adjustment Dyads), After Adjustment of Protocol (Post-Adjustment Dyads) and in Dyads From MMU.

The majority of errors were made in dyads that followed the protocol prior to the adjustments. Adjusting the written instruction and letting assessors view the videos twice led to significantly fewer errors being made, with a medium to large effect size (see Table 3). One-way ANOVA with Tukey as post hoc test to correct for multiple comparisons revealed that the UiO dyads in the pre-adjustment group (*n* = 12) made significantly more errors (*M* = 48.7, *SD* = 26.1) than both the UiO post-adjustment dyads (*n* = 8, *M* = 12.3, *SD* = 21.5) and the MMU dyads (*n* = 19, *M* = 21.3, *SD* = 18.3), *F*(2, 36) = 8.56, *p* < .001, η^2^ = .32 (Figure 6). The pre-adjustment group made as many errors using PAS (*M* = 23.8, *SD* = 20.8) as EP (*M* = 25.0, *SD* = 18.3), *t*(11) = –0.14, *p* = .889, *d* = .04, and compared to the other two groups, the number of errors was higher in the pre-adjustment group both with PAS, *F*(2, 36) = 3.51, *p* = .040, η^2^ = .16, and with EP, *F*(2, 36) = 7.02, *p* = .003, η^2^ = .28.

**Table 3:** Categories With Errors and Total Number of Errors Made in Dyads (n = 39) Related to Before and After Adjustment of Written Instruction, Mode of Instruction, Type of Access, and the Use of Graphic Symbols With PAS

	Errors made in dyads during test administration			
Variable	Mean (*SD*)	Mean (*SD*)	Mean (*SD*)	Sign
*Adjustment of original protocol*				
	Before adjustment	After adjustment		
	(*n* = 12 dyads)	(*n* = 27 dyads)		
Categories with errors	5.6 (2.1)	4.4 (2.0)		*t*(37)^a^ = 1.74, *p* = .045*, *d* = 0.60
Number of errors	48.7 (26.1)	18.6 (19.3)		*t*(37)^a^ = 4.02, *p* < .001*, *d* = 1.39
*First instruction mode*				
	Written instruction	Video instruction	Both written and video instruction	
	(*n* = 18)	(*n* = 15)	(*n* = 6)	
Categories with errors	4.9 (1.8)	4.2 (2.2)	5.4 (2.4)	*F*(2, 36)^b^ = 0.86, *p* = .432, η^2^ = 0.05
Number of errors	33.7 (27.3)	23.1 (24.6)	22.5 (22.2)	*F*(2, 36)^b^ = 0.85, *p* = .435, η^2^ = 0.05
*Order of access*				
	PAS first, EP last	EP first, PAS last		
	(*n* = 19)	(*n* = 20)		
Categories with errors	4.7 (2.2)	4.8 (1.9)		*t*(37)^c^ = 0.20, *p* = .984, η^2^ = 0.00
Number of errors	33.7 (28.1)	22.3 (22.1)		*t*(37)^c^ = –1.41, *p* = .166, η^2^ = 0.05
*PAS and graphic symbols*				
	Use of symbols	No use of symbols		
	(*n* = 20)	(*n* = 19)		
Categories with errors	2.4 (1.8)	2.2 (1.1)		*t*(37)^a^ = 0.24, *p* = .407, *d* = 0.08
Number of errors	17.8 (21.8)	9.4 (7.4)		*t*(37)^a^ = 1.60, *p* = .117, *d* = 0.51

a Independent samples t-tests, equal variances assumed, one-sided p. ^b^One-way ANOVA. ^c^Paired-samples *t*-tests.

* *p* < .05.

### Errors Related to Mode of Instruction and Access

There was no significant difference that could be related to whether the participants first got the written instruction, the video instruction, or both at the same time, although a trend could be observed toward less errors being made when a video instruction came first or simultaneously with a written instruction. There was no significant difference that could be related to whether they first used EP or PAS as access mode, albeit the results suggest that somewhat fewer errors were made when EP preceded PAS. Using symbols to answer “yes” and “no” with PAS led to more errors being made, as evidenced by a medium effect size, although the difference did not reach significance (see Table 3).

## Discussion

The aim of this study was to investigate the a) reliability of, b) transparency of, and c) adherence to protocols for assessments using the alternative response modalities PAS and EP.

The reliability of the protocol was investigated by comparing the number of errors recorded by the two observers, who did this independently of each other. An intraclass correlation coefficient of .97 between the two observers indicates high agreement. The mode of administration observed, PAS or EP, did not affect reliability.

Most novice practitioners managed to deliver the test according to the stated protocols, albeit with a small number of mistakes. There were more errors using PAS than EP, but this difference did not reach statistical significance. A finding that was significant, however, was that most mistakes were made in the dyads from UiO completing the assessments prior to the adjustments of the protocol, with a moderate to large effect size between the groups concerning number of errors made and categories with errors. There was a tendency toward the Norwegian dyads to complete the assessments post-adjustment making the fewest errors, but the difference between this group and the MMU dyads did not reach statistical significance. Together, the results therefore indicate that the final Norwegian and UK protocols are fairly transparent and easy to adhere to.

The written and video formats of the protocols worked just as well, noting that allowing for viewing the video twice (procedure at UiO post-adjustment) or together with the written instructions (MMU procedure for six dyads) led to fewer errors, even if the difference between the different formats did not reach significance.

As for adherence to the protocol, it was encouraging to observe that the majority of the assessors positioned themselves correctly, that is, beside the responder using PAS and opposite the responder using EP (see Figure 1 for an illustration). Another positive finding is that most assessors also placed the test material correctly. Both correct positioning and correctly placed test material are essential during EP, as it makes it possible for the assessor to accurately see where respondent is looking (Stadskleiv, 2015). Furthermore, we anticipated that the novice assessors might use a leading tone of voice when administering the test items, as this is a mistake that is quite easy to make. It is also an unfortunate mistake to make, as it might contribute to the person being assessed (the responders) getting a score that overestimates their cognitive abilities and consequently not receiving the right level of interventions. It was therefore encouraging to find that this mistake was not made frequently.

One type of mistake that occurred frequently during PAS was disruption. This might have been an artifact of the situation, where the students should “assess” the language comprehension of a fellow student on very easy items, while at the same time being observed by two fellow students (i.e., the observers). This may have influenced the students’ performance, and it seems reasonable to assume that this behavior would be quite different if they were to assess children or adults in a more realistic setting. On the other hand, failure to record examinee responses, which is an error frequently observed in studies of examiner errors during intelligence testing (Styck & Walsh, 2016), was rarely seen in our sample. We categorized this error as “Assessment scoring form not used correctly” in our study, and results showed it to be equally rare in both EP and PAS. One probable reason for this is that the record form for this task was simple to fill out, as the task had a multiple-choice format—compared to intelligence tests with open-ended questions where the examiner also has to write down the verbal responses of the examinee.

The other mistakes that were frequent, that is, “Instructions incomplete,” “Wrong phrasing of test question,” and “Incomplete procedure/sequence,” might be related to the initial Norwegian protocols not being precise enough. Still, it underlines the importance of always completing the whole sequence of test administration, especially when using PAS. Without completing the whole sequence, on all items, the responders might understand what the right or wrong answer to an item is, or be denied the opportunity to change his or her initial response.

In Norway, *YES* and *NO* symbols were provided and meant to be used in the PAS context. However, many of the novice practitioners did not use the symbols, as it felt more natural to answer orally than to point at symbols, and thus “wrong use of yes/no symbols” was one of the most common types of errors made using PAS. In a real assessment situation, this would probably be less of an issue, as the person being assessed will use the form of yes/no response that they are already familiar with. Many children and adults will either have their own graphic symbols for expressing *YES* and *NO*, or will have their own way of expressing yes/no that does not involve the use of graphic symbols, for example, looking to the right to express “yes” and to the left to express “no.” This was taken into account at MMU, where it was decided to not include this response condition to simulate a more typical response form of children and adults with severe motor and speech impairments, where you would find out which unaided responses they used for yes and no.

The assessors sometimes had leading gaze when assessing using EP. This mistake of looking in the direction of the correct answers could be from an interest in wanting to see where the correct answer was or simply that they were unaware of doing it. On the other hand, it could also be a result of not being familiar with the test and therefore not knowing that the key to any delivery is to maintain gaze with the responder until they select an item, at which point looking at the chosen option is part of confirming the responders’ choice. Nevertheless, this points to the importance of recognizing the aim of using an EP approach in assessment. The goal is to gather autonomous input and responses from the individual being assessed as a way of establishing their cognitive and linguistic levels. By contrast, this might be compared with the use of EP during other forms of interaction where a co-constructed approach to message generation and a rapid response may be the primary aim. The challenge with such approaches is to differentiate between what is the autonomous contribution of the individual and what is co-constructed with the facilitator.

### Limitations and Strengths

There are some limitations to this study. First, a priori power calculations indicated that we needed a sample size of 32 dyads, and we managed to include 39. However, of these 39, 12 dyads could be considered a pilot group in the sense that they followed the pre-adjustment protocols. Thus, we only have 27 dyads that followed the final protocol, making the sample size marginally smaller than planned. Nevertheless, the sample is big enough to indicate that the final alternative response mode protocols work. Another limitation is that this study includes three slightly different procedures.

The use of novice practitioners that are fellow students might have affected the execution of the tests. We asked the responders to role-play being children to remove any stress the responders might have about being “assessed.” However, this might have made the situation seem less real for the students, especially for those from UiO who only assessed fellow students posing as children. The number of disruptions during the assessment that occurred suggests as much and must also be considered a limitation of the study design. At MMU, it was professionals who role-played being children. Although this may on one hand have contributed positively toward the student assessors taking the assessment situation more seriously, on the other hand, it might also have added slightly more pressure to them, as novice practitioners. This should also be noted as a limitation of the study.

It might also be seen as a limitation that the assessor administered both PAS and EP to the same respondent, as it is typical that persons using AAC employ only one of these as an access method to their communication material (von Tetzchner et al., 2025). However, in clinical practice, one might encounter clients for whom it would be beneficial to be able to switch between the response modes PAS and EP, depending on factors such as the layout of test material and fatigue resulting from using one response mode for some time.

The strengths of this study are many. We systematically gave EP and PAS instructions in a different order. EP given first and PAS given second resulted in fewer mistakes both in type and frequency. This might have been because the EP protocol was clearer and more straightforward when it comes to the execution of the test. There was also less material to handle, for example, PAS also included the use of *YES* and *NO* symbols in Norway. The protocol given in video format also led to these results. This might be a result of the protocol providing more detailed visual information when describing PAS.

### Clinical Implications

There is still room for improvement in the instructional material, but this study indicates that it is feasible for novice assessment practitioners to administer tests using EP and PAS, and that it is possible to do so using paper-based test material that the clinician already has access to. This has important clinical implications, as it documents the possibility of conducting standardized testing with individuals who have severe speech and motor impairments. The findings of this study offer enhanced possibilities for investigating an individual’s language and cognitive potential. The study confirms that alternative modes of responding to test items can be successfully taught to novice practitioners, and it seems reasonable to expect that the protocols therefore will also be easily understood by more experienced professionals. In the future, it is to be hoped that this might lead to more individuals with severe speech and motor impairments being offered comprehensive assessment, and as a consequence of that interventions that are better tailored to their linguistic abilities and cognitive functioning.

Furthermore, as the protocol was administered both in Norwegian and English, it seems reasonable to assume that it can be successfully translated into other languages and used in diverse clinical settings. Further studies should investigate the adherence to alternative response mode protocols when assessing children and clinical populations and compare the use of such protocols with standardized assessment procedures.

## Supplemental Material

sj-docx-1-asm-10.1177_10731911251315012 – Supplemental material for Assessment of Cognition and Language Using Alternative Response Modalities Supplemental material, sj-docx-1-asm-10.1177_10731911251315012 for Assessment of Cognition and Language Using Alternative Response Modalities by Kristine Stadskleiv, Katy Latham, Kristina Tufteskog Spanne, Karen Sætre, Anna Fraas, Ilaria Ruscito, Yasmine Taha and Janice Murray in Assessment

sj-docx-2-asm-10.1177_10731911251315012 – Supplemental material for Assessment of Cognition and Language Using Alternative Response Modalities Supplemental material, sj-docx-2-asm-10.1177_10731911251315012 for Assessment of Cognition and Language Using Alternative Response Modalities by Kristine Stadskleiv, Katy Latham, Kristina Tufteskog Spanne, Karen Sætre, Anna Fraas, Ilaria Ruscito, Yasmine Taha and Janice Murray in Assessment
