# Supplementary material for: Assessment of Cognition and Language Using Alternative Response Modalities
Source: Assessment. 2025 Feb 12;33(1):46–58. doi: 10.1177/10731911251315012 (PMC12686183; doi:10.1177/10731911251315012)
Supplement: sj-docx-1-asm-10.1177_10731911251315012 – Supplemental material for Assessment of Cognition and Language Using Alternative Response Modalities [file sj-docx-1-asm-10.1177_10731911251315012.docx]

**Instruction for Eye Pointing as answer method on test**

When administering this test, it is important that you do not give any hints to the person assessed about which answer is correct. For example, use a neutral tone of voice when pointing out the four answer options. Look directly at the person being assessed, not at the picture showing the correct answer option. For each task, show the person tested that there are four answer options. Do not name the pictures. Present the tasks at the eye height of the person. Keep a distance of appr. 1 meter.

Here is an example of how to perform a task:

**Target word: Strawberry. Correct answer: Alternative B.**

Hold the task sheet at eye height, so that you can see the person assessed through the transparent sheet. Say «*I want you to look at all these pictures*». Point to each of the four pictures in sequence (A to D) with approximate 1 sec pause between each and say «*this*» (point to A), «*this*» (B), «*this*» (C) and «*this*» (D). Say «*Now I want you to look at the picture showing the word I am saying. Where is the strawberry*?».


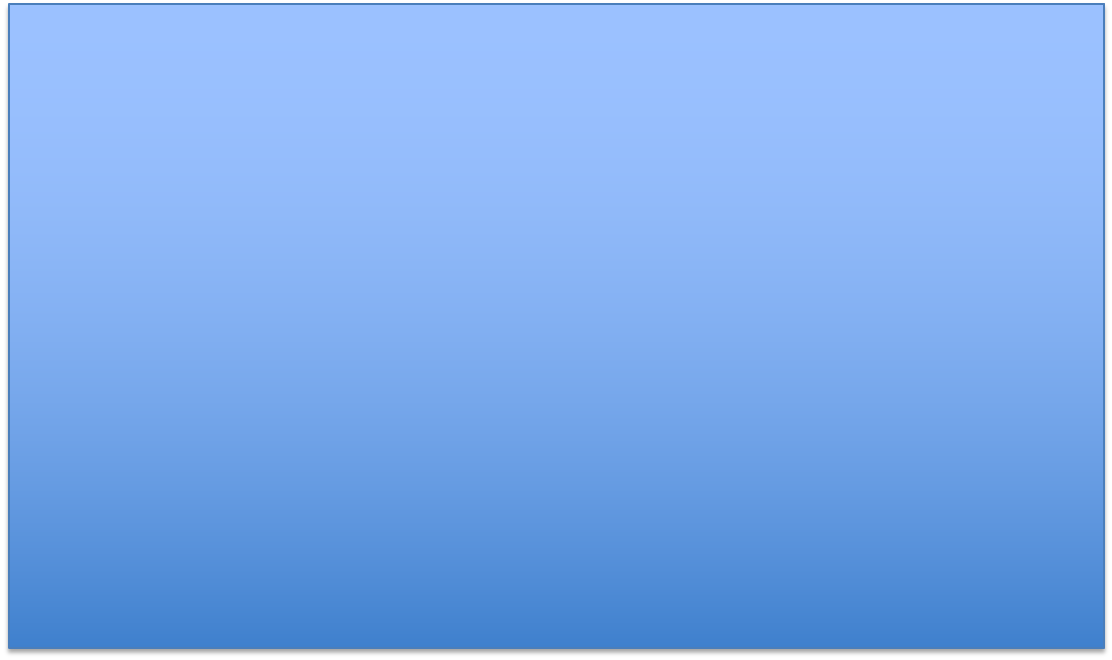


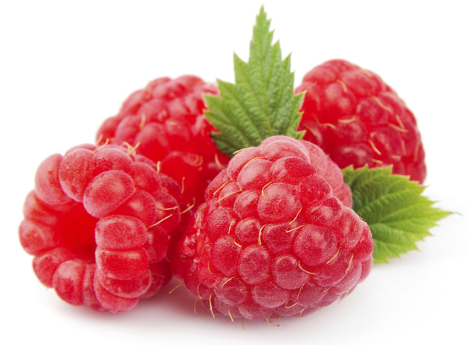

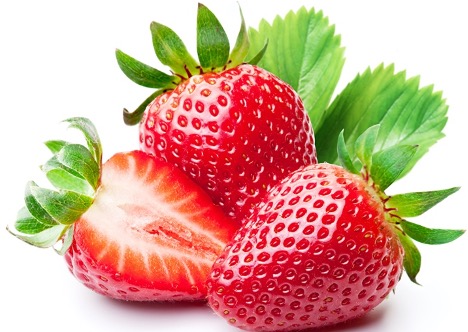


*A B*


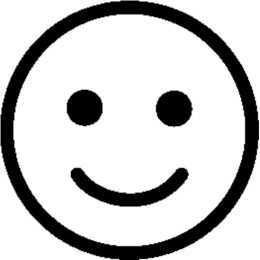


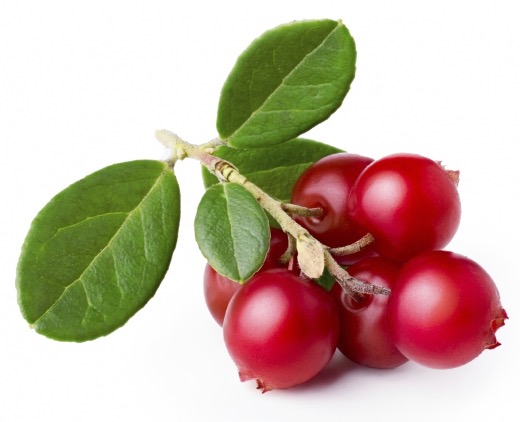

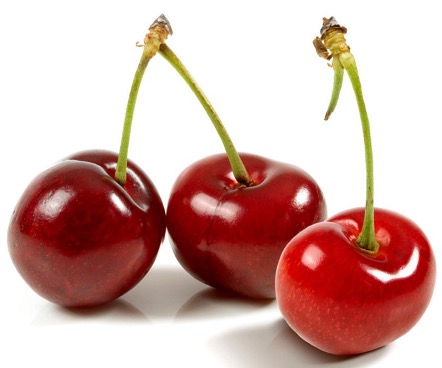


*C D*

On task 1, it is possible to repeat the procedure one time if the person does not respond or answer incorrectly. With no response, repeat the procedure If the person answers incorrectly, say, *« I think you looked at this one* (point to A, C or D), *but that is not quite correct. Let’s try again*». Repeat the instruction. On all other tasks, note down the response (A through D or zero response) and proceed to the next.
