# Supplementary material for: Assessment of Cognition and Language Using Alternative Response Modalities
Source: Assessment. 2025 Feb 12;33(1):46–58. doi: 10.1177/10731911251315012 (PMC12686183; doi:10.1177/10731911251315012)
Supplement: sj-docx-2-asm-10.1177_10731911251315012 – Supplemental material for Assessment of Cognition and Language Using Alternative Response Modalities [file sj-docx-2-asm-10.1177_10731911251315012.docx]

**Instruction for Partner Assisted Scanning as answer method on test**

When administering this test, it is important that you do not give any hints to the person assessed about which answer is correct. For example, use a neutral tone of voice when pointing out the four answer options. For each task, show the person tested that there are four answer options. Do not name the pictures. Ask the person assessed to show you how they say *yes* and *no.* The person assessed should use the included graphical symbols *YES* and *NO* (see below) and answer by pointing.

Here is an example of how to perform a task:

**Target word: Strawberry. Correct answer: Alternative B.**

Hold up the task sheet in front of the person and sit down next to the person. Say «*I want you to look at all these pictures*». Point to each of the four pictures in sequence (A to D) with approximate 1 sec pause between each and say «*this*» (point to A), «*this*» (B), «*this*» (C) and «*this*» (D). Say «*Now we shall look at each of these four pictures, and you should express YES and NO to indicate if the word I am saying is on that picture.”*  Say “*Where is the strawberry? Is it this (*point to A), *is it this (*point to B), *is it this* (point to C), *is it this?* (point to D).” Note on the registration form the answer option for which the person indicates *YES.* Complete the whole sequence (all four pictures) for all tasks, regardless of which answer option the person’s answers YES to and whether that is correct.

**
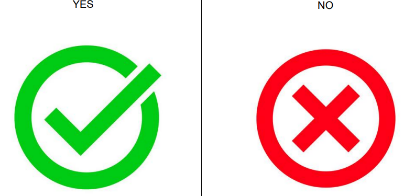
**

| **A**  **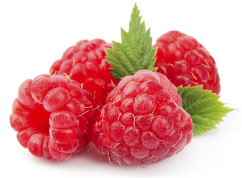** | **B** 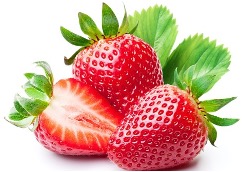 |
| --- | --- |
| **C**  **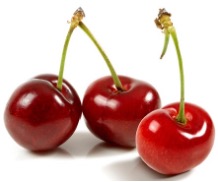** | **D**  **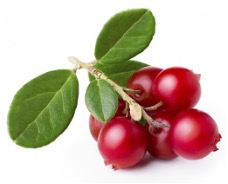** |

On the first task (task 21), it is possible to repeat the procedure once if the person does not respond or answer incorrectly. With no response, repeat the procedure. If the person answers incorrectly, say, «*I think you answered YES to this one* (point to A, C or D)*, but that is not quite correct. Let’s try again*». Repeat the instruction. On all other tasks, note down the response (A through D or zero response) and proceed to the next.
